# Regime-Switching Discrete ARMA Models for Categorical Time Series

**DOI:** 10.3390/e22040458

**Published:** 2020-04-17

**Authors:** Christian H. Weiß

**Affiliations:** Department of Mathematics and Statistics, Helmut Schmidt University, 22043 Hamburg, Germany; weissc@hsu-hh.de; Tel.: +49-40-6541-2779

**Keywords:** categorical time series, discrete ARMA models, parsimonious Markov chain, regime-switching models

## Abstract

For the modeling of categorical time series, both nominal or ordinal time series, an extension of the basic discrete autoregressive moving-average (ARMA) models is proposed. It uses an observation-driven regime-switching mechanism, leading to the family of RS-DARMA models. After having discussed the stochastic properties of RS-DARMA models in general, we focus on the particular case of the first-order RS-DAR model. This RS-DAR(1) model constitutes a parsimoniously parameterized type of Markov chain, which has an easy-to-interpret data-generating mechanism and may also handle negative forms of serial dependence. Approaches for model fitting are elaborated on, and they are illustrated by two real-data examples: the modeling of a nominal sequence from biology, and of an ordinal time series regarding cloudiness. For future research, one might use the RS-DAR(1) model for constructing parsimonious advanced models, and one might adapt techniques for smoother regime transitions.

## 1. Introduction

Since the pioneering textbook on time series analysis by Box & Jenkins [1], this topic has attracted an immense interest in research and applications. To put it more precisely, *real-valued* time series (having a range consisting of real numbers or vectors) have been in the limelight of scientists and practitioners since that time. Besides many approaches for analyzing real-valued time series, also enumerable models have been developed, starting with the basic autoregressive moving-average (ARMA) models [1]. ARMA models are characterized by a linear conditional mean and an autocorrelation function (ACF) satisfying the so-called Yule–Walker equations. Their stochastic properties are well understood, but their actual potential for application is limited. Therefore, many alternatives and extensions have been developed, which cover more realistic time series patterns [2]. For example, if being concerned with a time series exhibiting sudden jumps and a piecewise behavior, then it is more appropriate to consider regime-switching models like the self-exciting threshold (SET) AR models proposed by Tong & Lim [3], also see the survey in Tong [4].

During the last decades, *discrete-valued* time series received more and more attention, see Weiß [5] for a recent survey. Especially count time series, i.e., quantitative time series with a range included in the set $N_{0}=\left\{0,1,\ldots \right\}$ of non-negative integers, have been studied intensively. A large number of models have been developed for this type of discrete data, not only integer-valued counterparts to the basic ARMA model, but also to more advanced models like the aforementioned SETAR models. The latter include the proposals by Möller [6], Möller et al. [7], Monteiro et al. [8], Thyregod et al. [9], Wang et al. [10].

In this article, we consider another type of discrete-valued time series, which is somewhat neglected in the time series literature: *categorical* time series $x_{1},\ldots ,x_{T}$ with T∈N={1,2,…} and a qualitative range S consisting of a finite number of categories [5]. There are fundamental differences between quantitative scales (interval or ratio) and qualitative scales (ordinal or nominal) of measurement, see Table 1 in Stevens [11]. In particular, quantitative scales (such as the aforementioned count data, or real-valued measurements such as temperatures or prices) allow us to use the basic arithmetic operations, which, however, is not permitted for qualitative scales. Qualitative (categorical) ranges S are further distinguished into the cases that S exhibits a natural order among the categories (*ordinal* range), and that the categories in S are unordered (*nominal* range). In what follows, we are interested in both types of categorical time series. To simplify notations, we always assume the possible outcomes to be arranged in a certain order (either lexicographical or natural order), i.e., we denote the range as $S=\{s_{0},s_{1},\ldots ,s_{m}\}$ with some m∈N. Categorical time series require a tailor-made treatment in any sense. All the moment-based tools developed for quantitative time series cannot be applied due to the inadmissibility of the basic arithmetic operations. Instead, we have to express the location in terms of the mode (for ordinal time series, also the median can be used), and to use one of the customized measures of dispersion and serial dependence from Table 1, see Klein et al. [12], Weiß [13], [14] for further details. Even the basic time series plot can only be done in the ordinal case, whereas we may use the rate evolution graph as a substitute for nominal time series [5].

Finally, the selection of possible models for categorical time series is yet limited, see Weiß [5]. The quite flexible, higher-order Markov models suffer from a huge number of model parameters, which increases exponentially in the model order and polynomially in the number of categories, m+1. The latter might be reduced by amalgamating some categories, but this causes a loss of information. The extremely parsimonious discrete ARMA models by Jacobs & Lewis [15], in contrast, have a rather narrow scope of application, see the discussion in Section 2 for further details. Therefore, as a compromise between flexibility and parsimony, we extend the discrete ARMA models for categorical time series by an observation-driven regime-switching mechanism, see Section 3. For ordinal time series, it might be implemented in analogy to the SET approach for quantitative time series. However, the regime-switching can also be applied to nominal time series to capture, e.g., structures or similarities within the categorical range. As an important special case, we obtain a family of parsimonious Markov chain (MC) models, see Section 4. The application potential of the new model family is demonstrated in Section 5 with two real-data applications, where we also illustrate how model fitting might be done. Finally, we conclude in Section 6 and outline issues for future research.

## 2. About Discrete ARMA Models

Jacobs & Lewis [15] proposed two families of discrete ARMA models. The first of these families (labeled by the acronym “DARMA”) is defined in a nested way and has therefore found less attention in the literature. The second family (labeled as “NDARMA”), in contrast, directly imitates the ordinary ARMA recursion and has been considered in several subsequent works. Both families agree in their boundary cases (i.e., pure AR- and pure MA-type models). We concentrate on the “NDARMA family” in the sequel, and we refer to these models simply as discrete ARMA models. The regime-switching mechanism to be proposed in Section 3 could be applied to the “DARMA family” in an analogous way. According to Weiß & Göb [16], the discrete ARMA models (“NDARMA”) by Jacobs & Lewis [15] might be defined as follows (Jacobs & Lewis [15] provide an equivalent definition based on backshift operators).

**Definition** **1.** *Let ${\left(X_{t}\right)}_{Z}$ and ${\left(\epsilon_{t}\right)}_{Z}$ be categorical processes with range S, where ${\left(\epsilon_{t}\right)}_{Z}$ is independent and identically distributed (i. i. d.) with marginal distribution **p** ($\epsilon_{t}∼p$), and where $\epsilon_{t}$ is independent of ${\left(X_{s}\right)}_{s<t}$. Let*

$$D_{t}\;=\;\left(\alpha_{t,1},\ldots ,\alpha_{t,p},\beta_{t,0},\ldots ,\beta_{t,q}\right)\;∼\;\mathrm{Mult}\left(1;\;\rho \right)$$

*be i. i. d. multinomial random vectors with $\rho =(\phi_{1},\ldots ,\phi_{p},\varphi_{0},\ldots ,\varphi_{q})$, which are independent of ${\left(\epsilon_{t}\right)}_{Z}$ and of ${\left(X_{s}\right)}_{s<t}$. So the probabilities in ρ, $\phi_{1},\ldots ,\varphi_{q}\in \left(0;1\right)$, sum up to one.*

*Then, ${\left(X_{t}\right)}_{Z}$ is said to be a* discrete ARMA(p,q) process* if it follows the recursion*

(1) $$X_{t}\;=\;\alpha_{t,1}\cdot X_{t-1}+\ldots +\alpha_{t,p}\cdot X_{t-p}\;+\;\beta_{t,0}\cdot \epsilon_{t}+\ldots +\beta_{t,q}\cdot \epsilon_{t-q}.$$

*(Here, if the range S is not numerically coded, then we assume 0·s=0, 1·s=s and s+0=s for each s∈S.)*

The boundary cases q=0 and p=0 are referred to as a *DAR(p) process* and *DMA(q) process*, respectively. The probability vector ***p*** is contained in the (m+1)-part unit simplex (recall that the range S consists of m+1 categories),

$$S_{m+1}\;=\;\left\{u\in {\left(0;1\right)}^{m+1}\;|\;u_{0}+\ldots +u_{m}=1\right\},$$

and leads to *m* model parameters. Analogously, $\rho \in S_{p+q+1}$ leads to p+q further model parameters. Based on a Markov-chain representation of the discrete ARMA(p,q) process according to Definition 1, Weiß [17] concluded on the ergodicity of the process as well as on the existence of a unique stationary distribution. The initial distribution for achieving stationarity is obtained by solving the invariance equation corresponding to the Markov-chain representation.

Although being denoted in an “ARMA style”, the model recursion of Equation (1) implies that $X_{t}$ is generated by doing nothing else than simply selecting either one of the past p observations $X_{t-1},\ldots ,X_{t-p}$, or one of the available q+1 innovations $\epsilon_{t},\ldots ,\epsilon_{t-q}$. As a consequence, $X_{t}$ and $\epsilon_{t}$ have the same stationary marginal distribution, namely *p*, i.e., $P\left(X_{t}=s_{i}\right)\;=\;p_{i}\;=\;P\left(\epsilon_{t}=s_{i}\right)$ for all i=0,…,m. Furthermore, the random-selection mechanism leads to the following transition probabilities:

(2) $$\begin{array}{c} P(X_{t}=i_{0}\;|\;X_{t-1}=i_{1},\ldots X_{t-p}=i_{p},\epsilon_{t}=j_{0},\epsilon_{t-1}=j_{1},\ldots ,\epsilon_{t-q}=j_{q})\\ \;=\;\sum_{r=1}^{p}\delta_{i_{0}i_{r}}\phi_{r}\;+\;\delta_{i_{0}j_{0}}\varphi_{0}\;+\;\sum_{r=1}^{q}\delta_{i_{0}j_{r}}\varphi_{r}\\ \;=:\;p_{\left(p,q\right)}\left(i_{0}\;|\;i_{1},\ldots ,i_{p},\;j_{0},\ldots ,j_{q};\;\rho \right), \end{array}$$

where empty sums (in case of q=0 or p=0) are assumed to take the value 0. Here, $\delta_{i,j}$ denotes the Kronecker delta, which takes the value 1 (0) iff i=j (i≠j).

Despite their quite extraordinary data-generating mechanism, the discrete ARMA processes according to Definition 1 have an ARMA-like serial dependence structure. If serial dependence is expressed in terms of Cohen’s κ from Table 1, then the following Yule–Walker equations hold [16]:

(3) $$\kappa \left(h\right)\;=\;\sum_{j=1}^{p}\phi_{j}\;\kappa (|h-j|)\;+\;\sum_{i=0}^{q-h}\varphi_{i+h}\;r\left(i\right)\;\mathrm{for}\;h\geq 1,$$

where the r(i) are given by $r\left(i\right)\;=\;\sum_{j=\max\left\{0,i-p\right\}}^{i-1}\phi_{i-j}\cdot r\left(j\right)\;+\;\varphi_{i}\;1\left(0\leq i\leq q\right)$. Here, 1(A) denotes the indicator function, which takes the value 1 (0) iff *A* is true (false). Furthermore, Weiß [17] showed that the discrete ARMA processes are ϕ-mixing with exponentially decreasing weights. As a consequence, one can apply the central limit theorem on p. 200 in Billingsley [18] to establish the asymptotic normality for statistics derived from such a process.

**Remark** **1.** *Equation (3) also applies to the ordinal version of Cohen’s κ in Table 1. The reason for this is given by the fact that the bivariate probabilities at lag h, $p_{ij}\left(h\right)$, satisfy $p_{ij}\left(h\right)\;=\;\left(1-\kappa (h)\right)\;p_{i}p_{j}\;+\;\kappa \left(h\right)\;\delta_{i,j}\;p_{j}$ for discrete ARMA processes. As a result, one obtains $f_{ij}\left(h\right)\;=\;\left(1-\kappa (h)\right)\;f_{i}f_{j}\;+\;\kappa \left(h\right)\;f_{\min\{i,j\}}$ in the ordinal case, so $f_{ii}\left(h\right)-f_{i}^{2}\;=\;\kappa \left(h\right)\;f_{i}\left(1-f_{i}\right)$. Thus, for discrete ARMA processes, the identity $\kappa \left(h\right)=\kappa_{\mathrm{ord}}\left(h\right)$ has to hold, whereas these measures usually differ from each other for other data-generating processes. It should be pointed out that further identities with other measures of serial dependence exist, see Weiß [13], Weiß & Göb [16] for details.*

While the discrete ARMA models are very attractive in view of parameter parsimony (only m+p+q parameters) and some of its model properties (e.g., Yule–Walker equations for κ(h)), they suffer from the fact that only positive forms of serial dependence are possible (in the sense that always κ(h)≥0). Furthermore, because of the simple selection mechanism in Equation (1), the sample paths generated by discrete ARMA models are characterized by long constant segments being finished by abrupt changes, which will often be inappropriate for real applications. For quantitative time series, a possible remedy is to use additional variation operators, see Möller & Weiß [19]. However, this solution cannot be applied to qualitative time series. Therefore, in Section 3, the novel regime-switching discrete ARMA models are proposed to offer solutions to both of the above drawbacks, the limitation to positive dependence and the piecewise constant sample paths.

## 3. Regime-Switching Discrete ARMA Models

**Figure 1:**
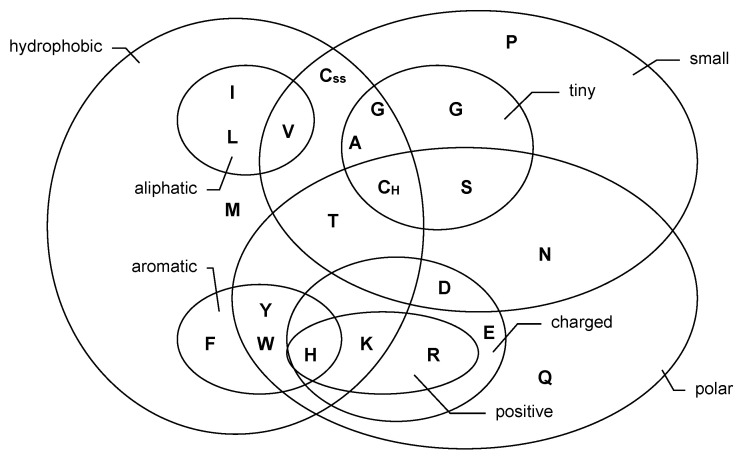
Venn diagram for the classification of amino acids, adapted from Figure 3a in Taylor [20].

Let ${\left(X_{t}\right)}_{Z}$ be a categorical process with range $S=\{s_{0},s_{1},\ldots ,s_{m}\}$. If the range S is ordinal, then the states are strictly ordered imposing a distinct structure on S. Even for a nominal range, the states are not necessarily free of any relations, but similarities might exist within it. An example is given by biological sequences such as deoxyribonucleic acid (DNA) and protein sequences. The four DNA bases ‘a’, ‘c’, ‘g’, and ‘t’ (adenine, cytosine, guanine, and thymine, respectively) are divided into the group of pyrimidines (c, t) and purines (a, g). In this sense, c is more similar to t than to a or g. The twenty different amino acids exhibit an even more refined similarity structure, which is visualized by the Venn diagram in Figure 1, see Taylor [20] for further details. If developing a stochastic model for a biological sequence (also see Section 5.1 below), it is reasonable to try to account for the apparent structure within the range.

As a possible solution for this task, let us now introduce a novel regime-switching (RS) extension of the discrete ARMA model. It is defined with respect to a partition of the range $S=\{s_{0},s_{1},\ldots ,s_{m}\}$ into *K* non-empty subsets, where 1≤K≤m+1. Here, $S_{1},\ldots ,S_{K}$ constitute a partition of S iff these sets are pairwise disjoint and satisfy $S=S_{1}\cup \ldots \cup S_{K}$. The *K* regimes shall be used as a means to account for a structure within the categorical range, such as the grouping of the DNA bases in pyrimidines and purines, or the ordering of the categories in the case of an ordinal time series. The current regime is determined by the last (or even earlier) observation: if $X_{t-1}\in S_{k}$, then the process is in the *k*th regime at time *t*, and the upcoming observation $X_{t}$ is generated according to a regime-specific model. So we are concerned with an observation-driven (“self-exciting”) RS-mechanism, which is in contrast to, e.g., the Hidden-Markov model [21], where the regimes are defined by a latent process. As an example, for the ordinal cloudiness time series to be discussed in Section 5.2, we consider (among others) a two-regime model (so K=2), where the “lower regime” refers to a sky with at most scattered clouds, and the “upper regime” to broken clouds or an even overcast sky. Being in the lower regime, the upcoming cloudiness state follows a different model as if being in the upper regime.

**Definition** **2.** *Like in Definition 1, let ${\left(X_{t}\right)}_{Z}$ and ${\left(\epsilon_{t}\right)}_{Z}$ be categorical processes, where the range S is partitioned into $S_{1},\ldots ,S_{K}$, and let $D_{t}=\left(\alpha_{t,1},\ldots ,\beta_{t,q}\right)$ denote the multinomial mixture vectors.*

*Let $p_{\epsilon }^{\left(1\right)},\ldots ,p_{\epsilon }^{\left(K\right)}\in S_{m+1}$ and let $\rho^{\left(1\right)},\ldots ,\rho^{\left(K\right)}\in S_{p+q+1}$ be K state-dependent probability vectors. Then, the regime-switching discrete ARMA(p,q) process (“RS-DARMA”) is defined by the recursive scheme*

(4) $$\begin{array}{cc} X_{t}\;= & \alpha_{t,1}\cdot X_{t-1}+\ldots +\alpha_{t,p}\cdot X_{t-p}\;+\;\beta_{t,0}\cdot \epsilon_{t}+\ldots +\beta_{t,q}\cdot \epsilon_{t-q}\\ \; & with\;D_{t}∼\mathrm{Mult}\left(1,\rho_{t}\right)\;\mathrm{and}\;\epsilon_{t}∼p_{t},\\ \; & where\;\rho_{t}=\rho^{\left(k\right)}\;\mathrm{and}\;p_{t}=p_{\epsilon }^{\left(k\right)}\;iff\;X_{t-1}\in S_{k}. \end{array}$$

Note that the boundary case K=1 leads to the ordinary discrete ARMA model. In Definition 2, we stated the most basic type of RS-condition, where the last observation $X_{t-1}$ determines the current regime. Certainly, one may use other conditions as well, e.g., based on more delayed observations $X_{t-d}$ with d>1.

If $S=S_{1}\cup \ldots \cup S_{K}$ denotes a partition, then each s∈S belongs to exactly one of the $S_{1},\ldots ,S_{K}$. To simplify notations, we introduce the mapping π:S→{1,…,K} defined by π(s)=k iff $s\in S_{k}$. So the element s∈S belongs to the π(s)-th subset. Then, the RS-condition in Equation (4) can be rewritten as $p_{t}=p_{\epsilon }^{\left(\pi \left(X_{t-1}\right)\right)}$ and $\rho_{t}=\rho^{\left(\pi \left(X_{t-1}\right)\right)}$.

Note that the number of possible partitions of a set of size m+1 into *K* subsets is equal to S(m+1,K), a Stirling number of the second kind [22], p. 5. These numbers can be computed recursively according to S(m+1,K)=K·S(m,K)+S(m,K−1) with S(0,0)=1 and S(m,0)=0=S(0,m). The total number of all partitions of S into non-empty subsets, $B_{m+1}=\sum_{K=1}^{m+1}S\left(m+1,K\right)$, is the (m+1)-th Bell number [22], p. 5.

**Example** **1.** *Recall the DNA example mentioned in the beginning of Section 3. There, the range consists of m+1=4 different states, S={a,c,g,t}. Since m=3, there are $B_{4}=15$ possible partitions, namely 1,7,6,1 different partitions into K=1,2,3,4 subsets, respectively. One of them splits into the purines and pyrimidines, i.e., S is partitioned into $S_{1}=\left\{a,g\right\}$ and $S_{2}=\left\{c,t\right\}$. In this particular case, π maps a↦1, c↦2, g↦1, and t↦2.*

Let us now discuss the stochastic properties of the RS-DARMA(p,q) process according to Definition 2. The ordinary discrete ARMA’s transition probabilities from Equation (2) change to

(5) $$\begin{array}{c} P(X_{t}=i_{0}\;|\;X_{t-1}=i_{1},\ldots X_{t-p}=i_{p},\epsilon_{t}=j_{0},\epsilon_{t-1}=j_{1},\ldots ,\epsilon_{t-q}=j_{q})\\ \;=\;p_{\left(p,q\right)}\left(i_{0}\;|\;i_{1},\ldots ,i_{p},\;j_{0},\ldots ,j_{q};\;\rho^{\left(\pi \left(i_{1}\right)\right)}\right), \end{array}$$

i.e., depending on the outcome $X_{t-1}=i_{1}$, another set of dependence parameters $\rho^{\left(\pi \left(i_{1}\right)\right)}$ is plugged-in into Equation (2), otherwise, we just have the ordinary discrete-ARMA structure. Therefore, the MC-representation of the discrete ARMA process as well as the resulting proofs for existence, ergodicity, and mixing properties (ϕ-mixing with exponentially decreasing weights), as provided by Section 2.2 in Weiß [17], can be adapted to the RS-DARMA process, see Appendix A for details.

**Example** **2.** *Let us consider the case q=0, i.e., the purely autoregressive RS-DARMA model according to Definition 2. It can be understood as the direct counterpart to the popular SETAR model. The RS-DAR(p) process constitutes a pth-order Markov process, where the transition probabilities compute as*

$$\begin{array}{c} P(X_{t}=s_{i}\;|\;X_{t-1}=s_{i_{1}},\ldots X_{t-p}=s_{i_{p}})\\ \;=\;\sum_{j=0}^{m}P\left(X_{t}=s_{i}\;|\;X_{t-1}=s_{i_{1}},\ldots X_{t-p}=s_{i_{p}},\epsilon_{t}=s_{j}\right)\;P\left(\epsilon_{t}=s_{j}\;|\;X_{t-1}=s_{i_{1}}\right)\\ \;\overset{\left(5\right)}{=}\;\sum_{j=0}^{m}p_{\left(p,q\right)}\left(s_{i}\;|\;s_{i_{1}},\ldots ,s_{i_{p}},\;s_{j};\;\rho^{\left(\pi \left(s_{i_{1}}\right)\right)}\right)\;p_{\epsilon ,j}^{\left(\pi \left(s_{i_{1}}\right)\right)}\\ \;\overset{\left(2\right)}{=}\;\sum_{j=0}^{m}\left(\sum_{r=1}^{p}\delta_{s_{i}s_{i_{r}}}\phi_{r}^{\left(\pi \left(s_{i_{1}}\right)\right)}\;+\;\delta_{s_{i}s_{j}}\varphi_{0}^{\left(\pi \left(s_{i_{1}}\right)\right)}\right)\;p_{\epsilon ,j}^{\left(\pi \left(s_{i_{1}}\right)\right)}\\ \;=\;\sum_{r=1}^{p}\delta_{s_{i}s_{i_{r}}}\phi_{r}^{\left(\pi \left(s_{i_{1}}\right)\right)}\;+\;\varphi_{0}^{\left(\pi \left(s_{i_{1}}\right)\right)}\;p_{\epsilon ,i}^{\left(\pi \left(s_{i_{1}}\right)\right)}. \end{array}$$

*These can now be used for likelihood computations or forecasting purposes.*

## 4. A Class of Parsimonious Markov Chains

A particularly important special case of the RS-DARMA family is obtained by setting (p,q)=(1,0), because such a RS-DAR(1) process constitutes a parsimoniously parameterized Markov chain (MC), also see Example 2. MCs, in turn, are of great relevance, because (1) such a first-order memory is often sufficient in practice, and because (2) MCs may constitute the starting point for defining more complex time series models [5]. Well-known examples regarding (2) are the so-called mixture transition distribution (MTD) model proposed by Raftery [23], which extends an underlying MC to a higher-order Markov model with only one additional parameter for each increment of the model order, or the hidden-Markov model (HMM), where the observable process is controlled by a latent MC [21]. Recall that an HMM can be interpreted as a parameter-driven RS-model, whereas we consider observation-driven RS-models in this article.

For any of the extensions in the sense of (2), it would be of relevance to start with a maximally parsimonious MC model to keep the overall number of model parameters at a feasible level. A full MC model on S has m(m+1) model parameters, which increases quadratically in *m*. The lower bound of model parameters is determined through the i. i. d.-case, where only the marginal distribution *p* has to be specified (which requires *m* parameters). So any non-i. i. d. model with unspecified marginal distribution must have ≥m+1 parameters. The ordinary DAR(1) model with its m+1 parameters reaches this lower bound (and also the so-called “Negative Markov model”, see, e.g., Weiß [13] for details). It may sometimes be too simplistic for practice, recall the discussion in Section 2. In fact, an MTD(p) model relying on a DAR(1)-MC just leads to a DAR(p) model. Thus, using a (true) RS-DAR(1) model as a base for defining a HMM or MTD model, respectively, might turn out as a reasonable compromise between model flexibility and parameter parsimony.

The model recursion of an ordinary DAR(1) process can be denoted as

(6) $$X_{t}\;=\;\alpha_{t}\;X_{t-1}\;+\;\left(1-\alpha_{t}\right)\;\epsilon_{t}\;\mathrm{with}\;\alpha_{t}∼\mathrm{Bin}\left(1,\phi \right),\;\epsilon_{t}∼p_{\epsilon },$$

where $\phi =\phi_{1}$ and $p_{\epsilon }=p$ according to Definition 1. Its transition probabilities equal $p_{i|j}=P\left(X_{t}=i\;|\;X_{t-1}=j\right)\;=\;\left(1-\phi \right)\;p_{\epsilon ,i}+\phi \;\delta_{i,j}$. Let $S=S_{1}\cup \ldots \cup S_{K}$ be a partition, then the *RS-DAR(1) model* is generally defined by

(7) $$X_{t}\;=\;\alpha_{t}\;X_{t-1}\;+\;\left(1-\alpha_{t}\right)\;\epsilon_{t}\;\mathrm{with}\;\alpha_{t}∼\mathrm{Bin}\left(1,\phi^{\left(\pi \left(X_{t-1}\right)\right)}\right),\;\epsilon_{t}∼p_{t}^{\left(\pi \left(X_{t-1}\right)\right)},$$

see Example 2. For applications, however, it might be better to impose further restrictions such that the resulting model is better interpretable. Therefore, we shall now propose two special cases of Equation (7), where the regimes affect either the marginal distribution or the dependence parameter.

### 4.1. Marginal Regimes

A *RS-DAR(1) model with respect to the marginals* is defined by

(8) $$\begin{array}{cc} X_{t}\;= & \alpha_{t}\;X_{t-1}\;+\;\left(1-\alpha_{t}\right)\;\epsilon_{t}\;\mathrm{with}\;\alpha_{t}∼\mathrm{Bin}\left(1,\phi \right),\;\epsilon_{t}∼p_{t},\\ \; & \mathrm{where}\;p_{t}=p_{\epsilon }^{\left(k\right)}\;\mathrm{iff}\;X_{t-1}\in S_{k},\;i.e.,\;p_{t}=p_{\epsilon }^{\left(\pi \left(X_{t-1}\right)\right)}. \end{array}$$

Here, $p_{\epsilon }^{\left(1\right)},\ldots ,p_{\epsilon }^{\left(K\right)}\in S_{m+1}$ are the *K* state-dependent probability vectors for the innovations $\epsilon_{t}$, implying Km+1 parameters for model Equation (8). K=m+1 would lead to a full MC model, but then ϕ would not be identifiable anymore. So model Equation (8) requires K≤m. The transition probabilities follow as $p_{i|j}\;=\;\left(1-\phi \right)\;p_{\epsilon ,i}^{\left(\pi (j)\right)}+\phi \;\delta_{i,j}$, see Example 2. In contrast to the ordinary DAR(1) model, the RS-DAR(1) model Equation (8) also allows for negative serial dependence. This can be obtained by choosing the $p_{\epsilon }^{\left(k\right)}$ such that $p_{\epsilon ,i}^{\left(\pi (i)\right)}\to 0$ for all i=0,…,m.

**Example** **3.** *Let m=3, and define the partition $S_{1}=\left\{s_{0},s_{1}\right\}$ and $S_{2}=\left\{s_{2},s_{3}\right\}$. Furthermore, let us consider the boundary case*

$$p_{\epsilon }^{\left(1\right)}\;=\;\left(0,0,\;p^{\left(1\right)},1-p^{\left(1\right)}\right){}^{⊤},\;p_{\epsilon }^{\left(2\right)}\;=\;\left(p^{\left(2\right)},1-p^{\left(2\right)},\;0,0\right){}^{⊤},$$

*where $p^{\left(1\right)},p^{\left(2\right)}\in \left(0;1\right)$. Then $p_{\epsilon ,i}^{\left(\pi (i)\right)}=0$ for all i=0,…,m. The transition matrix $P={\left(p_{i|j}\right)}_{i,j=0,\ldots ,m}$ equals*

$$P\;=\;\left(\begin{array}{cccc} \phi & 0 & \left(1-\phi \right)\;p^{\left(2\right)} & \left(1-\phi \right)\;p^{\left(2\right)}\\ 0 & \phi & \left(1-\phi \right)\;\left(1-p^{\left(2\right)}\right) & \left(1-\phi \right)\;\left(1-p^{\left(2\right)}\right)\\ \left(1-\phi \right)\;p^{\left(1\right)} & \left(1-\phi \right)\;p^{\left(1\right)} & \phi & 0\\ \left(1-\phi \right)\;\left(1-p^{\left(1\right)}\right) & \left(1-\phi \right)\;\left(1-p^{\left(1\right)}\right) & 0 & \phi \end{array}\right).$$

*Solving the invariance equation Pp=p, the stationary marginal distribution, $X_{t}∼p$, equals $p=\frac{1}{2}\;\left(p^{\left(2\right)},1-p^{\left(2\right)},p^{\left(1\right)},1-p^{\left(1\right)}\right){}^{⊤}$. From the diagonal of P, it becomes clear that the bivariate probabilities $p_{ii}\left(1\right)=\phi \;p_{i}$. Furthermore, $2\;\sum_{i=0}^{m}p_{i}^{2}=1-p^{\left(1\right)}\left(1-p^{\left(1\right)}\right)-p^{\left(2\right)}\left(1-p^{\left(2\right)}\right)$, so one computes Cohen’s κ at lag 1, see Table 1, as *

$$\kappa \left(1\right)\;=\;1\;-\;\frac{2\;(1-\phi )}{1+p^{\left(1\right)}\left(1-p^{\left(1\right)}\right)+p^{\left(2\right)}\left(1-p^{\left(2\right)}\right)}.$$

**Figure 2:**
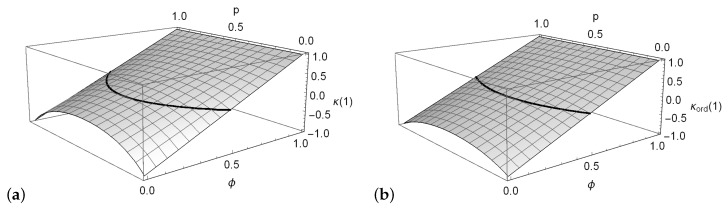
Example 3: Plot of (**a**) κ(1) and (**b**) $\kappa_{\mathrm{ord}}\left(1\right)$ against ϕ and p with $p^{\left(1\right)}=p^{\left(2\right)}=p$. The black curves indicate those (ϕ,p), where κ(1)=0 or $\kappa_{\mathrm{ord}}\left(1\right)=0$, respectively.

*This expression might also become negative, which is illustrated in Figure 2a, where κ(1) is plotted against ϕ and p with $p^{\left(1\right)}=p^{\left(2\right)}=p$.*

*Note that if the range is assumed ordinal, then $\kappa_{\mathrm{ord}}\left(1\right)$ differs from κ(1) (in contrast to the case of ordinary DAR(1) models). With analogous computations as before, one obtains*

$$\kappa_{\mathrm{ord}}\left(1\right)\;=\;1\;-\;\frac{2\;\left(1-\phi \right)\;\left(2+p^{\left(2\right)}-p^{\left(1\right)}\right)}{2+p^{\left(2\right)}-p^{\left(1\right)}\;+\;p^{\left(1\right)}\left(1-p^{\left(1\right)}\right)+p^{\left(2\right)}\left(1-p^{\left(2\right)}\right)}.$$

*But like for κ(1), also $\kappa_{\mathrm{ord}}\left(1\right)$ might take negative values, see Figure 2b.*

### 4.2. Dependence Regimes

As a possible alternative to Equation (8), a *RS-DAR(1) model with respect to the dependence parameter* is defined by

(9) $$\begin{array}{cc} X_{t}\;= & \alpha_{t}\;X_{t-1}\;+\;\left(1-\alpha_{t}\right)\;\epsilon_{t}\;\mathrm{with}\;\alpha_{t}∼\mathrm{Bin}\left(1,\phi_{t}\right),\;\epsilon_{t}∼p_{\epsilon },\\ \; & \mathrm{where}\;\phi_{t}=\phi^{\left(k\right)}\;\mathrm{iff}\;X_{t-1}\in S_{k},\;i.e.,\;\phi_{t}=\phi^{\left(\pi \left(X_{t-1}\right)\right)}. \end{array}$$

Here, $\phi^{\left(1\right)},\ldots ,\phi^{\left(K\right)}\in \left(0;1\right)$ are the *K* state-dependent dependence parameters, so model Equation (9) has altogether m+K parameters. The transition probabilities compute as $p_{i|j}\;=\;\left(1-\phi^{\left(\pi (j)\right)}\right)\;p_{\epsilon ,i}+\phi^{\left(\pi (j)\right)}\;\delta_{i,j}$, see Example 2.

**Example** **4.** *As a possible application of model Equation (9), consider an ordinal range S with m≥2, and let us assume individual dependence parameters for each state. More precisely, let us assume the partition $S={⋃}_{k=0}^{m}S_{k}$ with $S_{k}=\left\{s_{k}\right\}$, and the corresponding m+1 state-dependent dependence parameters $\phi^{\left(0\right)},\ldots ,\phi^{\left(m\right)}\in \left(0;1\right)$. So we have 2m+1 model parameters, whereas a full MC would have m(m+1) parameters. The transition probabilities equal $p_{i|j}=\left(1-\phi^{\left(j\right)}\right)\;p_{\epsilon ,i}+\phi^{\left(j\right)}\;\delta_{i,j}$. Then $X_{t}∼p$ with*

$$p_{j}\;=\;\frac{\frac{p_{\epsilon ,j}}{1-\phi^{\left(j\right)}}}{\sum_{l=0}^{m}\frac{p_{\epsilon ,l}}{1-\phi^{\left(l\right)}}},\;and\;\;p_{ij}\;=\;p_{i|j}\;p_{j}\;=\;\frac{p_{\epsilon ,i}\;p_{\epsilon ,j}+\delta_{i,j}\;\frac{\phi^{\left(i\right)}}{1-\phi^{\left(i\right)}}\;p_{\epsilon ,i}}{\sum_{l=0}^{m}\frac{p_{\epsilon ,l}}{1-\phi^{\left(l\right)}}}.$$

*The formula for $p_{i}$ is easily verified by computing*

$$\sum_{j=0}^{m}p_{ij}\;=\;\frac{p_{\epsilon ,i}\;+\;\frac{\phi^{\left(i\right)}}{1-\phi^{\left(i\right)}}\;p_{\epsilon ,i}}{\sum_{l=0}^{m}\frac{p_{\epsilon ,l}}{1-\phi^{\left(l\right)}}}\;=\;\frac{\frac{p_{\epsilon ,i}}{1-\phi^{\left(i\right)}}}{\sum_{l=0}^{m}\frac{p_{\epsilon ,l}}{1-\phi^{\left(l\right)}}}.$$

*The cumulative probabilities f of $X_{t}$ are given by $f_{i}=\sum_{j=0}^{i}\frac{p_{\epsilon ,j}}{1-\phi^{\left(j\right)}}/\sum_{l=0}^{m}\frac{p_{\epsilon ,l}}{1-\phi^{\left(l\right)}}$. It follows that*

$$p_{ii}\;=\;\frac{\frac{p_{\epsilon ,i}}{1-\phi^{\left(i\right)}}-p_{\epsilon ,i}\;\left(1-p_{\epsilon ,i}\right)}{\sum_{l=0}^{m}\frac{p_{\epsilon ,l}}{1-\phi^{\left(l\right)}}},\;f_{ii}\;=\;\sum_{r,s=0}^{i}p_{rs}\;=\;\frac{\sum_{j=0}^{i}\frac{p_{\epsilon ,j}}{1-\phi^{\left(j\right)}}\;-f_{\epsilon ,i}\;\left(1-f_{\epsilon ,i}\right)}{\sum_{l=0}^{m}\frac{p_{\epsilon ,l}}{1-\phi^{\left(l\right)}}},$$

*which can be used to compute the dependence measures κ(1) and $\kappa_{\mathrm{ord}}\left(1\right)$ from Table 1.*

### 4.3. Statistical Inference

Let θ denote the vector of all model parameters, i.e., for the RS-DAR(1) model Equation (8), we have $\theta =\left(\phi ,\;p_{\epsilon ,1}^{\left(1\right)},\ldots ,p_{\epsilon ,m}^{\left(K\right)}\right)\in {\left(0;1\right)}^{K\;m+1}$, whereas $\theta =\left(\phi^{\left(1\right)},\ldots ,\phi^{\left(K\right)},\;p_{\epsilon ,1},\ldots ,p_{\epsilon ,m}\right)\in {\left(0;1\right)}^{K+m}$ for model Equation (9). To estimate θ from a given time series $x_{1},\ldots ,x_{T}$, we use the maximum likelihood (ML) approach. The (conditional) ML estimate $\hat{\theta }$ is obtained by numerically maximizing the log-likelihood function,

(10) $$\ell \left(\theta \right)\;=\;\sum_{t=2}^{T}\ln p_{x_{t}|x_{t-1}}\left(\theta \right).$$

We denote the maximized log-likelihood by $\ell_{\max}=\frac{T}{T-1}\;\ell \left(\hat{\theta }\right)$, where the factor $\frac{T}{T-1}$ corrects for the conditioning on $x_{1}$ [5], p. 236. The existence, consistency, and asymptotic normality are easily established by proving that Condition 5.1 in Billingsley [24] holds. This condition requires that

1. the set $D=\left\{\left(k,l\right)\;|\;p_{k|l}\left(\theta \right)>0\right\}$ does not dependent on θ;
2. each $p_{k|l}\left(\theta \right)$ has continuous partial derivatives in θ;
3. the Jacobian matrix of ${\left(\ldots ,\partial p_{k|l}\left(\theta \right),\ldots \right)}_{(k,l)\in D}$ has full rank, i.e., the rank $n_{\mathrm{model}}=\dim\left(\theta \right)$;
4. the MC is irreducible.

Since the transition probabilities are quadratic polynomials in the model parameters, part 2 is always satisfied. Part 1 holds by restricting the model parameters to the open interval (0;1), then all $p_{k|l}\left(\theta \right)>0$. This also implies the irreducibility of the transition matrix. Part 3 is ensured by an appropriate design of the model (identifiability of parameters).

Finally, if the model design is not fixed by the considered application scenario, then one will commonly try out multiple types of partitioning (between the boundary cases of an ordinary DAR(1) model and a full MC). In this case, the model selection might be done based on a certain type of information criterion, such as Akaike’s information criterion (AIC) or the Bayesian information criterion (BIC), see Burnham & Anderson [25]. These are given by

(11) $$\mathrm{AIC}\;=\;-2\;\ell_{\max}\;+\;2\;n_{\mathrm{model}},\;\mathrm{BIC}\;=\;-2\;\ell_{\max}\;+\;n_{\mathrm{model}}\;\ln T,$$

respectively. The performance of AIC and BIC if selecting among general Markov models was investigated by Katz [26]. It was shown that only the BIC is consistent while the AIC tends to overfitting.

## 5. Real-Data Applications

In what follows, we apply the RS-DAR(1) models to two data examples. The first one refers to a DNA sequence, which constitutes a nominal time series (Section 5.1). The second example, in contrast, is about an ordinal time series of cloudiness states (Section 5.2).

### 5.1. DNA Sequence Modeling

**Figure 3:**
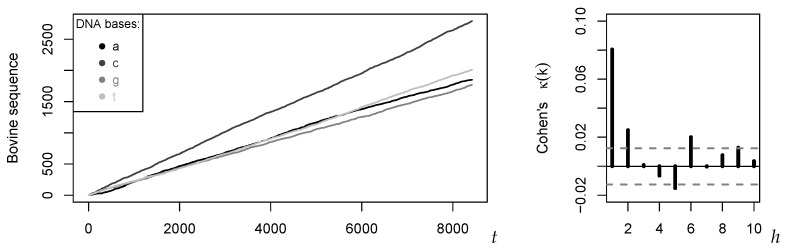
Rate evolution graph of Bovine sequence and of $\hat{\kappa }\left(h\right)$ against lag *h*.

Let us pick up the discussion in the beginning of Section 3 as well as in Example 1, where we considered a nominal DNA sequence having the range S={a,c,g,t} (so m=3). Although such a sequence does not constitute a “time” series in the original sense, it is common practice to use models for stochastic processes as a tool for summarizing its main properties, see Churchill [27], Dehnert et al. [28]. In what follows, we consider the DNA sequence of the Bovine leukemia virus (length T=8419), which is published by the National Center for Biotechnology Information at https://www.ncbi.nlm.nih.gov/nuccore/NC_001414?%3Fdb=nucleotide. Its rate evolution graph (a time series plot is not possible for nominal data, see Weiß [5]) and its sample Cohen’s κ (recall Table 1) are plotted in Figure 3.

The Bovine sequence was comprehensively analyzed in Section 7.2 of Weiß [5], where several types of models were fitted to the data. The most parsimonious model used was the ordinary DAR(1) model (four parameters), but the best fit was actually obtained by a full MC model (12 parameters). Weiß [5] also tried out a 2-state HMM (eight parameters), which improved over the DAR(1) model but was inferior to the full MC. Nevertheless, it is worth noting that the two hidden states that were constructed during model fitting closely matched the purines (a, g) and pyrimidines (c, t), respectively. This indicates a RS-approach might turn out to be appropriate for the data.

Therefore, we now fit several types of RS-DAR(1) model to these data. As the criterion for model selection, we use the BIC, which is very suitable for time series of large length *T*. First, we try out the approach of Section 4.2, where the DAR(1)’s dependence parameter ϕ depends on the current regime. The obtained BIC results are summarized in Table 2. The RS-DAR(1) model with two regimes (purines $S_{1}$ and pyrimidines $S_{2}$) and, thus, five parameters, leads to a slight improvement compared to the DAR(1) model, but the four-regime model (seven parameters) performs even worse in terms of the BIC. Any of these models is much worse than the full MC, so a regime-dependent dependence structure does not seem to be appropriate for the data.

Hence, let us now use the RS-DAR(1) model with regime-dependent innovations’ distribution, see Section 4.1. Starting again with the two-regime model (purines vs. pyrimidines; seven parameters), we achieve a considerable improvement over the DAR(1) model, but still do not reach the full MC’s BIC, see Table 3. Thus, we try splitting either the purine or the pyrimidine regime, leading to a three-regime model with ten parameters. If we split the purines into $S_{1}=\left\{a\right\},S_{2}=\left\{g\right\}$, then the BIC deteriorates again. However, if splitting the pyrimidines into $S_{2}=\left\{c\right\},S_{3}=\left\{t\right\}$, then the BIC improves and even becomes better than that of the full MC. So this type of RS-DAR(1) model is best among all candidate models.

Thus, let us analyze this model fit in some more detail. The dependence parameter is estimated as $\hat{\phi }\approx 0.061$, and the three regime-dependent innovations’ distributions are

$$\begin{array}{c} {\hat{p}}_{\epsilon }^{\left(1\right)}\approx {\left(0.244,0.299,0.245,0.211\right)}^{⊤},\;{\hat{p}}_{\epsilon }^{\left(2\right)}\approx {\left(0.216,0.352,0.143,0.289\right)}^{⊤},\\ {\hat{p}}_{\epsilon }^{\left(3\right)}\approx {\left(0.181,0.360,0.240,0.219\right)}^{⊤}. \end{array}$$

This implies, for example, that if $X_{t-1}\in S_{3}=\left\{t\right\}$, then the probability for $\epsilon_{t}\in S_{2}=\left\{c\right\}$ is quite large, whereas $X_{t-1}\in S_{1}=\left\{a,g\right\}$ also leads to a rather large probability for $\epsilon_{t}\in S_{1}$. These “transition rules” can also be seen from the resulting transition matrix

$$P_{\mathrm{fit}}\;\approx \;\left(\begin{array}{cccc} 0.291 & 0.203 & 0.229 & 0.170\\ 0.281 & 0.392 & 0.281 & 0.338\\ 0.230 & 0.134 & 0.292 & 0.225\\ 0.198 & 0.271 & 0.198 & 0.267 \end{array}\right),$$

which implies the following stationary marginal distribution for $X_{t}$:

$$p_{\mathrm{fit}}\;\approx \;{\left(0.220,0.331,0.210,0.239\right)}^{⊤}.$$

For the given three-digit rounding, this perfectly agrees with the vector of relative frequencies computed from the data. This excellent agreement between fitted and observed marginal distribution carries over to the measures of dispersion given in Table 1, with an IQV value of ≈0.988 and an entropy of ≈0.987. Both values are very close to 1, because the marginal distribution is quite close to a uniform distribution, which is considered as the maximally dispersed scenario for nominal data [13].

Finally, let us do a diagnostic check of the serial dependence structure. On p. 146 in Weiß [5], it was pointed out that the sample value of Cohen’s κ at lag 1, $\hat{\kappa }\left(1\right)\approx 0.080$, deviates from the corresponding sample value of Cramer’s *v*, $\hat{v}\left(1\right)\approx 0.113$. Here, Cramer’s *v* is defined by $v\left(k\right)={\left(\frac{1}{m}\;\sum_{i,j\in S}\;{\left(p_{ij}\left(k\right)-p_{i}p_{j}\right)}^{2}/\left(p_{i}p_{j}\right)\right)}^{1/2}$, constituting a so-called “unsigned” measure of serial dependence [5]. This discrepancy between $\hat{\kappa }\left(1\right)$ and $\hat{v}\left(1\right)$ contradicts a DAR(1) model, where κ(h) and v(h) exactly agree, but it was reproduced by the fitted full MC. For the fitted three-regime model, we obtain $\kappa_{\mathrm{fit}}\left(1\right)\approx 0.080$ and $v_{\mathrm{fit}}\left(1\right)\approx 0.111$. The fitted RS-DAR(1) model reproduces the discrepancy between κ and *v*, confirming its adequacy for the Bovine data.

### 5.2. Cloudiness Time Series

**Figure 4:**
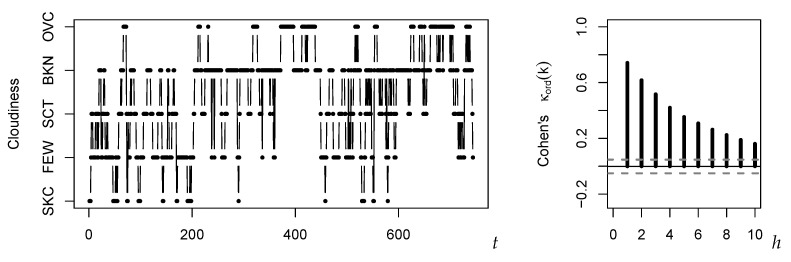
Plot of cloudiness time series and of ${\hat{\kappa }}_{\mathrm{ord}}\left(h\right)$ against lag *h*.

The amount of cloud coverage is measured in “okta”, i.e., in eighths of the sky being covered by clouds. Then, a common classification of cloudiness consists of the following five ordinal states (so m=4), ordered from lowest to highest: ‘SKC’ (sky clear, 0 oktas), ‘FEW’ (few, 1–2 oktas), ‘SCT’ (scattered, 3–4 oktas), ‘BKN’ (broken, 5–7 oktas), and  ‘OVC’ (overcast, 8 oktas). We considered a time series obtained from the “DWD Climate Data Center” offered by the Deutscher Wetterdienst (German Weather Service) at https://cdc.dwd.de/portal/201912031600/mapview. The data refer to the hourly observations of cloudiness at the weather station in Schleswig (a town in the north of Germany) in May 2011. So the time series is of total length T=744. A plot of the data as well as the corresponding sample ordinal Cohen’s κ (recall Table 1) are shown in Figure 4. We have a rather strong degree of serial dependence ($\hat{\kappa }\left(1\right)\approx 0.741$). The marginal cumulative frequencies are given by $\hat{f}\approx {\left(0.050,0.253,0.433,0.825\right)}^{⊤}$, leading to the dispersion values 0.626 for the sample IOV and 0.689 for the CPE (recall Table 1). Note that in the ordinal case, maximal dispersion does not go along with a uniform distribution, but with an extreme two-point distribution, i.e., $f={\left(0.5,\ldots ,0.5\right)}^{⊤}$.

In analogy to Section 5.1, we now fit types of MC model to the cloudiness data. The most parsimonious model is again the DAR(1) model, but this does not account for the ordinal structure of the range. Therefore, besides the full MC model, we also considered RS-DAR(1) models (with respect to the marginal distribution), which are designed such that they account for the natural order within the range (the RS-DAR(1) model with m+1 dependence regimes, see Example 4, performs clearly worse and is, thus, not reported here). This is achieved by imitating the threshold approach for quantitative time series: we considered the partitioning $S_{1}=\left\{\mathrm{SKC},\mathrm{FEW},\mathrm{SCT}\right\}$, $S_{2}=\left\{\mathrm{BKN},\mathrm{OVC}\right\}$ corresponding to a threshold at SCT, and the refinement $S_{1}=\left\{\mathrm{SKC},\mathrm{FEW}\right\}$, $S_{2}=\left\{\mathrm{SCT}\right\}$, $S_{3}=\left\{\mathrm{BKN},\mathrm{OVC}\right\}$ corresponding to two thresholds at FEW,SCT. The BICs of the four candidate models are summarized in Table 4.

According to the BIC, we select the two-regime model, where the dependence parameter is estimated as $\hat{\phi }\approx 0.547$, and where the two regime-dependent innovations’ distributions are

$$\begin{array}{cc} {\hat{p}}_{\epsilon }^{\left(1\right)}\;\approx & {\left(0.091,0.395,0.238,0.269,0.006\right)}^{⊤},\\ {\hat{p}}_{\epsilon }^{\left(2\right)}\;\approx & {\left(0.000,0.050,0.195,0.560,0.194\right)}^{⊤}. \end{array}$$

**Figure 5:**
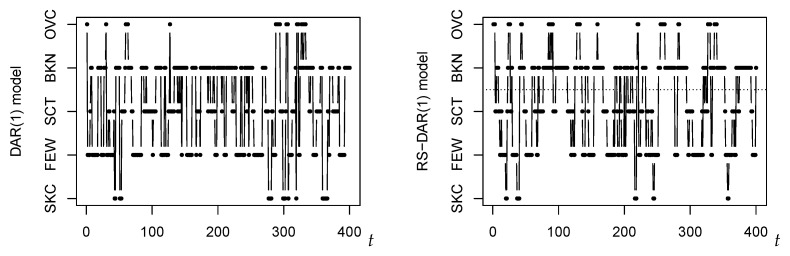
Plots of simulated cloudiness time series, generated according to the fitted DAR(1) model (**left**) and two-regime RS-DAR(1) model ((**right**); regimes separated by dotted line), respectively.

According to this fitted model, we mainly produce innovations from the lower regime if staying in the lower regime, and vice versa. This type of “inertia” can also be seen from the right part of Figure 5, where a time series was simulated according to the fitted two-regime model. This sample path looks much more similar to the original time series in Figure 4 than the simulated DAR(1) path in the left part of Figure 5, which does not exhibit a piecewise behavior. The (cumulative) stationary marginal distribution of the fitted two-regime model results as

$$f_{\mathrm{fit}}\;\approx \;{\left(0.043,0.256,0.471,0.894\right)}^{⊤},$$

which is reasonably close to $\hat{f}$. In fact, looking at the corresponding dispersion measures, we get ${\mathrm{IOV}}_{\mathrm{fit}}\approx 0.575$ and ${\mathrm{CPE}}_{\mathrm{fit}}\approx 0.640$, which is only slightly below the above sample values. The dependence structure is captured quite well, with $\kappa_{\mathrm{ord},\;\mathrm{fit}}\left(1\right)\approx 0.697$.

**Remark** **2.** *Since T=744 is not that large, one might also think of using the AIC for model selection. In this case, the three-regime model (AIC≈1290.1) would be preferred over the two-regime model (AIC≈1304.0). The parameter estimates are $\hat{\phi }\approx 0.592$ and*

$$\begin{array}{cc} {\hat{p}}_{\epsilon }^{\left(1\right)}\;\approx & {\left(0.154,0.414,0.302,0.130,0.000\right)}^{⊤},\\ {\hat{p}}_{\epsilon }^{\left(2\right)}\;\approx & {\left(0.031,0.486,0.001,0.464,0.018\right)}^{⊤},\\ {\hat{p}}_{\epsilon }^{\left(3\right)}\;\approx & {\left(0.000,0.055,0.204,0.361,0.380\right)}^{⊤}. \end{array}$$

*It is interesting to note that the innovations’ distribution of the central regime, $S_{2}=\left\{\mathrm{SCT}\right\}$, will hardly produce the value SCT itself. Instead, there is a nearly fifty-fifty chance of falling either above or below this value. The four additional model parameters are outweighed by a closer fit of the marginal distribution, now*

$$f_{\mathrm{fit}}\;\approx \;{\left(0.052,0.297,0.490,0.802\right)}^{⊤}$$

*with stronger dispersion (${\mathrm{IOV}}_{\mathrm{fit}}\approx 0.666$, ${\mathrm{CPE}}_{\mathrm{fit}}\approx 0.722$), and by more serial dependence $\kappa_{\mathrm{ord},\;\mathrm{fit}}\left(1\right)\approx 0.739$.*

The fitted two-regime model can now be applied to forecasting the cloudiness. The one-step-ahead conditional mode or median is always equal to the given state, i.e., the point forecast for the next hour is equal to the current cloudiness state. But if using 95% prediction intervals for weather forecasting, then more complex rules of the form “$X_{t}=a\;\Rightarrow \;X_{t+1}\in B$” are obtained:

$$\begin{array}{c} \mathrm{SKC}\;\Rightarrow \;\{\mathrm{SKC},\mathrm{FEW},\mathrm{SCT},\mathrm{BKN}\},\;\mathrm{FEW}\;\mathrm{or}\;\mathrm{SCT}\;\Rightarrow \;\{\mathrm{FEW},\mathrm{SCT},\mathrm{BKN}\},\\ \mathrm{BKN}\;\mathrm{or}\;\mathrm{OVC}\;\Rightarrow \;\{\mathrm{SCT},\mathrm{BKN},\mathrm{OVC}\}. \end{array}$$

## 6. Conclusions

We extended the basic discrete ARMA model of Jacobs & Lewis [15] by an observation-driven regime-switching mechanism, leading to the family of RS-DARMA models. Particular attention was given to two instances of the RS-DAR(1) model, because they constitute an easy-to-interpret type of parsimoniously parameterized MC model. Furthermore, in contrast to the ordinary DAR(1) model, the RS-DAR(1) model may even handle negative forms of serial dependence. Model fitting was illustrated by two real-data examples: a nominal DNA sequence, and an ordinal time series of cloudiness states. Besides such an immediate application of the novel models, it was also pointed out that types of RS-DAR(1) model might serve as the base for constructing parsimonious advanced models, such as MTD models or HMMs. This direction deserves further attention by future research. Furthermore, the special case of regime-switching ordinal processes, which has various applications in practice, should be further elaborated. Due to the close connection to bounded-counts time series [14], one may try to adapt regime-switching techniques known from count processes, e.g., hysteresis zones for enabling smoother regime transitions.

## Figures and Tables

**Table 1:** Some measures of dispersion and serial dependence for categorical time series.

	Nominal Range	Ordinal Range
**Dispersion**	*Index of qualitative variation* (*Gini index*):	*Index of ordinal variation*:
	$\mathrm{IQV}\;=\;\frac{m+1}{m}\;\left(1-\sum_{i=0}^{m}p_{i}^{2}\right)$,	$\mathrm{IOV}\;=\;\frac{4}{m}\;\sum_{i=0}^{m-1}f_{i}\left(1-f_{i}\right)$,
	*Entropy*:	*Cumulative paired entropy*:
	$\mathrm{En}\;=\;\frac{-1}{\ln(m+1)}\;\sum_{i=0}^{m}p_{i}\;\ln p_{i}$;	$\mathrm{CPE}\;=\;\frac{-1}{m\;\ln2}\;\sum_{i=0}^{m-1}\;\left(f_{i}\;\ln f_{i}+\left(1-f_{i}\right)\;\ln\left(1-f_{i}\right)\right)$;
	where $p_{i}=P\left(X=s_{i}\right)$, $p={\left(p_{0},\ldots ,p_{m}\right)}^{⊤}$	where $f_{i}=P\left(X\leq s_{i}\right)$, $f={\left(f_{0},\ldots ,f_{m-1}\right)}^{⊤}$
**Serial dependence**	*Cohen’s κ*:	*Ordinal Cohen’s κ*:
	$\kappa \left(h\right)\;=\;\frac{\sum_{i=0}^{m}\left(p_{ii}\left(h\right)-p_{i}^{2}\right)}{1-\sum_{i=0}^{m}p_{i}^{2}}$,	$\kappa_{\mathrm{ord}}\left(h\right)\;=\;\frac{\sum_{i=0}^{m-1}\left(f_{ii}\left(h\right)-f_{i}^{2}\right)}{\sum_{i=0}^{m-1}f_{i}\left(1-f_{i}\right)}$,
	where	where
	$p_{ij}\left(h\right)=P\left(X_{t}=s_{i},X_{t-h}=s_{j}\right)$ for time lag h∈N	$f_{ij}\left(h\right)=P\left(X_{t}\leq s_{i},X_{t-h}\leq s_{j}\right)$ for time lag h∈N

**Table 2:** Bovine DNA data: Bayesian information criterion (BIC) of RS-DAR(1) models with respect to dependence parameter ϕ, compared to those of ordinary DAR(1) and Markov chain (MC) model, respectively.

Model	DAR(1)	$S_{1}=\left\{a,g\right\},$	$S_{1}=\left\{a\right\},S_{2}=\left\{g\right\},$	Full MC
		$S_{2}=\left\{c,t\right\}$	$S_{3}=\left\{c\right\},S_{4}=\left\{t\right\}$	
BIC	22927.4	22926.3	22928.9	22824.6

**Table 3:** Bovine DNA data: BICs of RS-DAR(1) models with respect to marginals $p_{\epsilon }$, compared to those of ordinary DAR(1) and MC model, respectively.

Model	DAR(1)		$S_{1}=\left\{a\right\},$	$S_{1}=\left\{a,g\right\},$	Full MC
		$S_{1}=\left\{a,g\right\},$	$S_{2}=\left\{g\right\},$	$S_{2}=\left\{c\right\},$	
		$S_{2}=\left\{c,t\right\}$	$S_{3}=\left\{c,t\right\}$	$S_{3}=\left\{t\right\}$	
BIC	22927.4	22869.6	22875.0	22822.0	22824.6

**Table 4:** Cloudiness data: BICs of RS-DAR(1) models with respect to marginals $p_{\epsilon }$, compared to those of ordinary DAR(1) and MC model, respectively.

Model	DAR(1)		$S_{1}=\left\{\mathrm{SKC},\mathrm{FEW}\right\},$	Full MC
		$S_{1}=\left\{\mathrm{SKC},\mathrm{FEW},\mathrm{SCT}\right\},$	$S_{2}=\left\{\mathrm{SCT}\right\},$	
		$S_{2}=\left\{\mathrm{BKN},\mathrm{OVC}\right\}$	$S_{3}=\left\{\mathrm{BKN},\mathrm{OVC}\right\}$	
BIC	1423.4	1345.5	1350.1	1392.6

## Appendix A. Markov-Chain Representation

In analogy to the argumentation in Section 2.2 of Weiß [17], the RS-DARMA(p,q) process can be represented as the homogeneous MC ${\left(Z_{t}\right)}_{Z}$ defined by

(A1) $$Z_{t}\;:=\;{\left(X_{t};\;X_{t-1},\ldots ,X_{t-p+1},\;\epsilon_{t},\ldots ,\epsilon_{t-q+1}\right)}^{⊤}.$$

For q=0, no ϵ-terms need to be included in $Z_{t}$, but $X_{t}$ always has to be part of the vector $Z_{t}$. The corresponding transition probabilities are

(A2) $$\begin{array}{c} P\left(Z_{t}=\left(i_{0},\ldots ,i_{p-1},j_{0},\ldots ,j_{q-1}\right)\;|\;Z_{t-1}=\left(i_{1}^{\prime },\ldots ,i_{p}^{\prime },j_{1}^{\prime },\ldots ,j_{q}^{\prime }\right)\right)\\ \;=\;P(X_{t}=i_{0}\;|\;X_{t-1}=i_{1}^{\prime },\ldots X_{t-p}=i_{p}^{\prime },\epsilon_{t}=j_{0},\epsilon_{t-1}=j_{1}^{\prime },\ldots ,\epsilon_{t-q}=j_{q}^{\prime })\\ \;\;\cdot \;P(\epsilon_{t}=j_{0}\;|\;X_{t-1}=i_{1}^{\prime })\\ \;\;\cdot \;P\left(X_{t-1}=i_{1}\;|\;X_{t-1}=i_{1}^{\prime }\right)\cdots p\left(\epsilon_{t-q+1}=j_{q-1}\;|\;\epsilon_{t-q+1}=j_{q-1}^{\prime }\right)\\ \;=\;p_{j_{0}}^{\left(\pi \left(i_{1}^{\prime }\right)\right)}\cdot \delta_{i_{1}i_{1}^{\prime }}\cdots \delta_{j_{q-1}j_{q-1}^{\prime }}\cdot p_{\left(p,q\right)}\left(i_{0}\;|\;i_{1}^{\prime },\ldots ,i_{p}^{\prime },\;j_{0},j_{1}^{\prime },\ldots ,j_{q}^{\prime };\;\rho^{\left(\pi \left(i_{1}^{\prime }\right)\right)}\right), \end{array}$$

see Equation (5). The crucial point in the derivations of Weiß [17] is the Lemma in Appendix A.1, where it is shown that all (p+q+1)-step-ahead transition probabilities of ${\left(Z_{t}\right)}_{Z}$ are truly positive. From this property, it follows that ${\left(Z_{t}\right)}_{Z}$ is primitive, which implies the existence, ergodicity, and the mixing properties of ${\left(X_{t}\right)}_{Z}$.

For the RS-DARMA(p,q) model considered here, we compute

$$\begin{array}{cc} P(Z_{t}=a\;| & Z_{t-(p+q+1)}=b)\;=\;\sum_{\;\begin{array}{c} a_{\max\left\{p,1\right\}},\ldots ,a_{p+q},\\ b_{q},\ldots ,b_{p+q} \end{array}}\;\prod_{j=0}^{p+q}\\ \; & \;(P(X_{t-j}=a_{j}\;|\;X_{t-j-1}=a_{j+1},\ldots ,\epsilon_{t-j}=b_{j},\ldots )\\ \; & \;\cdot P(\epsilon_{t-j}=b_{j}\;|\;X_{t-j-1}=a_{j+1},\ldots ,\epsilon_{t-j-1}=b_{j+1},\ldots )) \end{array}$$

for all $a,b\in S^{\max\left\{p,1\right\}+q}$. The first factor follows from (5) as

$$\begin{array}{c} P(X_{t-j}=a_{j}\;|\;X_{t-j-1}=a_{j+1},\ldots ,\epsilon_{t-j}=b_{j},\ldots )\\ \;=\;p_{\left(p,q\right)}\left(a_{j}\;|\;a_{j+1},\ldots ,a_{j+p},\;b_{j},\ldots ,b_{j+q};\;\rho^{\left(\pi \left(a_{j+1}\right)\right)}\right), \end{array}$$

the second one is given by

$$P\left(\epsilon_{t-j}=b_{j}\;|\;X_{t-j-1}=a_{j+1},\ldots ,\epsilon_{t-j-1}=b_{j+1},\ldots \right)\;=\;p_{b_{j}}^{\left(\pi \left(a_{j+1}\right)\right)}.$$

The rest of the proof is done in the same way as in Appendix A.1 of Weiß [17], by using that all $p^{\left(k\right)}$ and $\rho^{\left(k\right)}$ have only non-zero entries.
